# Partial Submandibular Gland Resection With Ultrasonic Dissector vs Electrocautery

**DOI:** 10.1093/asjof/ojaf011

**Published:** 2025-02-11

**Authors:** Hallie Buckner, Anil R Shah

## Abstract

**Background:**

The superficial aspect of the submandibular gland can be removed to improve the aesthetic appearance of the face and neck. This study describes a novel surgical technique to resect part of the submandibular gland during a deep neck or facelift.

**Objectives:**

Describe the technique to remove the superficial lobe of the submandibular gland using an ultrasonic dissector. Analyze the association between the uses of ultrasonic technology and reduced postoperative complications. Compare rates of postoperative complications between standard electrocautery and ultrasonic dissection.

**Methods:**

Experimental and control groups were established. A Sonicision ultrasonic dissector (Covidien, Dublin, Ireland) was used for resection in the experimental group, whereas electrocautery was used for resection in the control group. Postoperative outcomes of neuropraxia, hematoma, seroma, and sialocele formation were collected. Statistical analysis was performed using a Fisher's exact test in RStudio.

**Results:**

Control patients (*n* = 32) experienced 1 hematoma, 3 seromas, 3 sialoceles, and 3 neuropraxias. Experimental patients (*n* = 48) experienced 2 seromas with no hematomas, sialoceles, or neuropraxia. No association of statistical significance between reduced risk of complications and use of ultrasonic dissection was found.

**Conclusions:**

This novel technique has the potential to improve the safety and efficacy of partial submandibular gland resection. However, a follow-up study with a greater sample size and without confounding variables, such as intraoperative injection of Botulinum toxin, is necessary.

**Level of Evidence: 3:**

(Therapeutic) 
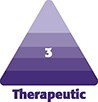

Partial submandibular gland removal can be used to improve the appearance of the face and neck in neck lifts and other aesthetic procedures.^1^ The submandibular gland is 1 of the 3 major types of salivary glands. After the parotid, it is the second largest salivary gland and produces mixed serous and mucous secretions.^2^ Saliva is produced in these glands and is secreted into the mouth through Wharton's duct, spanning 5 cm from the anterior submandibular gland to the frenulum of the tongue.^3^

Anatomically, the submandibular gland is located bilaterally in the region of the submandibular triangle. The submandibular triangle is bordered anteriorly and posteriorly by the digastric muscle. It is bound inferiorly by the hyoglossus muscle posteriorly and the mylohyoid muscle anteriorly. The submandibular gland is located beneath this muscle (Figure 1).^3^ It is surrounded by a capsule of soft tissue, consisting of 2 layers of investing deep fascia.^4^ The vascular supply of the submandibular gland stems from the facial artery, which is located on the posterior surface of the gland. There are 1 to 3 glandular branches supplying the submandibular gland that are subject to ligation during partial gland removal.^5^ These supplying branches may be the facial artery, submental artery, deep lingual artery, lingual artery, external carotid artery, or ascending palatine artery.^6,7^ Three nerves are located in close proximity to the submandibular gland: the lingual nerve, hypoglossal nerve, and facial nerve. The lingual nerve is intertwined with the gland and can be found laterally, inferiorly, and medially. Along with the submandibular ganglion, it is superficial to the submandibular gland, while the hypoglossal nerve is deep to the gland. The facial nerve can be found laterally.^2^ Any surgery involving this region must involve meticulous navigation to avoid damaging these delicate structures.

**Figure 1.**
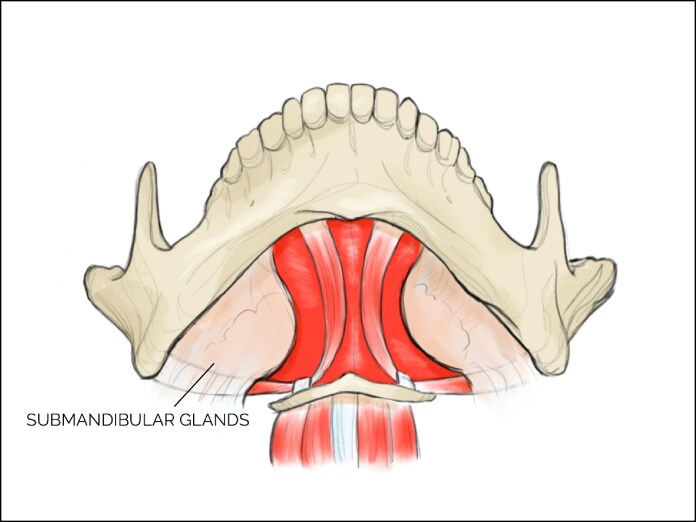
Anatomy of the submandibular gland. The gland is indicated by the text “submandibular gland.”

Although it is anatomically 1 lobe, the submandibular gland is often referred to as having superficial and deep lobes, with the dividing line being the mylohyoid muscle.^2^ It is the superficial lobe of the gland that is removed during aesthetic procedures to eliminate excess volume in the jaw and neck.^8^ This provides enhanced definition to both the lateral submandibular-cervical angle and jawline, drastically changing the appearance of the neck.^9,10^ However, submandibular gland resection carries the risk of serious complications, including bleeding in the deep neck spaces, temporary and permanent nerve damage, formation of seroma, and occurrence of sialocele.^11^

Of the possible side effects, the most significant is hematoma. Despite its low incidence (∼1%), these cases can quickly progress into surgical emergencies because of rapid accumulation of pressure in a closed space. Careful surgical management of surrounding vasculature is critical to avoid negative effects of hematoma, such as airway compression. It is often the deep perforator artery, usually originating from the facial or lingual arteries, that is transected during gland reduction and can result in hematoma.^4,12^ In addition to patient age, gender, BMI, comorbidities, and medications, ensuring proper hemostasis is critical in hematoma prevention.^13^

Seromas are a common complication that may occur following any surgical procedure or soft-tissue injury. They are often found following the creation of dead space in an operation. There are many proposed techniques for seroma prevention, including the intraoperative placement of quilting sutures. Once a seroma has formed, treatment ranges from massage therapy to surgical tunneling and can last several months.^14^

Currently, it is standard to use bipolar or monopolar electrocautery for submandibular gland removal.^12^ Although electrocautery can be helpful to temporarily stop immediate bleeding, studies have shown that the ultrasonic dissector may be best suited for this and other head and neck surgeries. Electrocautery reaches high temperatures, increasing the risk of damage to surrounding tissue, in addition to the potential for char dislodgement. Ultrasonic dissection lacks these high temperatures, using vibration to simultaneously cut and seal vessels.^15-18^ This tool was initially developed for use in abdominal or gastrointestinal procedures but has recently been adapted for use in a variety of head and neck surgeries.^19^ It consists of a 3-piece system that includes a handpiece, a reusable generator, and a reusable battery pack.^20^ It functions by vibrating at high frequency, producing frictional energy and heat between the instrument and tissue. This denatures proteins by breaking hydrogen bonds. When this is performed gradually and the temperature does not exceed 100 °C, denatured proteins form an insoluble gel that facilitates vessel coagulation.^21^ It is able to secure vessels up to 7 mm in diameter using this mechanism.^22^ To date, there have been no reported cases of partial submandibular gland removal using ultrasonic dissection. The aim of our study is to compare bipolar electrocautery to ultrasonic dissection for partial resections of the submandibular gland.

## METHODS

A single-center study was conducted. All patients undergoing procedures, including partial submandibular gland removal from 2022 to 2023 were included. This included all deep neck and facelift operations occurring at this center. This was the only inclusion criterion, and all cases were consecutive. Patients were separated into control and experimental groups. Patients in the control group underwent submandibular gland removal with the standard surgical technique using Bovie bipolar electrocautery. Ultrasonic dissection was used for partial resection of the submandibular gland in the experimental group.

At this center, use of ultrasonic dissection was incorporated into the standard protocol for partial submandibular gland resection beginning in January 2023. Thus, the experimental group consists of patients who underwent this procedure in 2023. To create the control group, the charts of the appropriate number of partial submandibular gland resection patients from 2022 were examined retrospectively.

All patients gave their informed consent for inclusion before study participation through an in-person, digital consent form. This form stated that their surgery results would be used in data analysis for the use of a new surgical device and technique. This study was conducted in accordance with ethical considerations, voluntary participation, informed consent, anonymity, and potential for harm.

### Surgical Technique

Preoperative ultrasound technology was used to assess individual submandibular gland size, ensuring greater intraoperative accuracy and safety.

Exposure was similar between groups. All patients underwent general anesthesia. An ∼3 cm incision was made in the patient's submental crease. Metzenbaum scissors were used for sharp dissection of the subcutaneous tissue from the platysma muscle. To form a platysma flap, the platysma was separated in the midline and suspended using Army–Navy retractors. The digastric muscles were then medialized to aid in visualization of the submandibular gland. Once identified, blunt dissection using Metzenbaum scissors took place in the subcapsular plane. Debakey forceps were then used to apply medial and inferior tension to the gland to expose the superficial lobe.

In the control group, a standard bipolar bayonet was used for coagulation during gland removal. Metzenbaum scissors were used for dissection and removal of the submandibular gland.

In the experimental group, a Sonicision (Covidien, Dublin, Ireland) dissector was used (Video). This was the only tool used for gland removal. The dissector was used for 5 s, with a subsequent period of 5 s allowed for heat dispersal. Using this technique, the gland was usually removed in 1 piece.

For all surgical patients, 10 units of Botulinum toxin were injected into the gland bilaterally to prevent saliva production following partial gland removal. The inferior aspect of the platysma flap was then dissected free from the underlying neck fascia. A small muscle resection took place at the inferior aspect of the platysma muscle, both with the bipolar bayonet and with the Sonicision dissector.

Postoperatively, patients had no dietary or sleep restrictions. No drains were placed during the procedure, and a 24 h light compressive garment was to be worn following surgery. Follow-up for all surgical patients was performed in the following intervals: 1 week, 6 weeks, 3 months, and 6 months.

### Analysis

Statistical analysis was performed using RStudio (2022.02.3; Posit, Boston, MA). A Fisher's exact test was used to compare postoperative outcomes of control and experimental groups. This statistical test was chosen because at least one of our expected counts was <5. A *P*-value less than the *α*-value of .05 was considered significant.

## RESULTS

**Figure 2.**
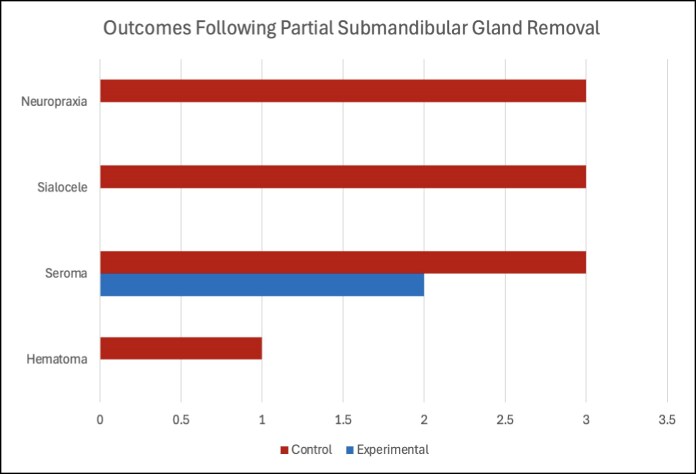
Postoperative outcomes for experimental and control patients. Control patients experienced 3 neuropraxias, 3 sialoceles, 3 seromas, and 1 hematoma. Experimental patients experienced 2 seromas and 0 neuropraxias, sialoceles, or hematomas.

The control group contained 32 patients, and the experimental group contained 48 patients for a total sample size of *n* = 80. The control group consisted of 7 male and 25 female patients, with an average age of 52.7 years. The minimum age for this group was 25 years, and the maximum was 75 years. The experimental group consisted of 11 male and 37 female patients, with an average age of 44.1 years. The minimum age for this group was 23 years, and the maximum was 76 years. Time for gland removal in the control and experimental groups was 30 and 15 min, respectively. Postoperatively, the control group had 1 hematoma (3.1%), 3 seromas (9.4%), 3 sialoceles (9.4%), and 3 occurrences of neuropraxia (temporary marginal mandibular nerve weakness) (9.4%). Comparatively, the experimental group had 2 seromas (4.2%) with no incidence of hematoma, sialocele, or neuropraxia (Figure 2). Hematoma formation occurred in the submental area, and all were treated with needle aspiration.

A Fisher's exact test was used to assess the relationship between the postoperative variables of hematoma, seroma formation, sialocele formation, and neuropraxia in control and experimental groups. Analysis of hematoma formation between experimental and control groups resulted in a *P*-value of 1. Similarly, analysis of seroma formation between experimental and control groups resulted in a *P*-value of .9198. Similar results were found when sialocele formation and neuropraxia were examined. Analysis of sialocele formation between the experimental and control groups yielded a *P*-value of 1. The analysis of neuropraxia between experimental and control groups also gave a *P*-value of 1. For each of the 4 postoperative variables, the *P*-value exceeds the *α*-value of .05. For this reason, we fail to reject the null hypothesis that there is no association between use of the Sonicision and a decreased risk of complications.

## DISCUSSION

Submandibular gland removal for aesthetic purposes has increased in popularity since its introduction. However, this procedure's drawbacks include increased risk of bleeding in deep neck spaces, nerve injury, and formation of seromas and sialoceles.^11^ Utilization of ultrasonic dissection over standard bipolar cautery resulted in a quantitative decrease in hematoma, seroma, sialocele, and neuropraxia cases. However, no associations of statistical significance were found between the use of the Sonicision and diminished postoperative complications.

The literature tells a different story. Use of ultrasonic dissection technology has provided improved results in these areas when compared with standard bipolar cautery. When used in superficial parotidectomy, ultrasonic dissection provided a statistically favorable hemostasis compared with scalpel and cautery (*P* < .01).^15^ Similar results were found in a meta-analysis when this instrument was used in thyroidectomy (*P* < .00001).^17,23^ Ultrasonic dissection has also provided favorable hemostasis over electrocautery outside of head and neck surgery. When compared in modified radical mastectomy, ultrasonic dissection had statistically significant intraoperative hemostatic outcomes (*P* < .001).^24^ Ultrasonic dissection was associated with decreased risk of seroma formation in similar procedures. Patients undergoing mastectomy and implant-based reconstruction experienced statistically decreased risk of developing postoperative seroma if ultrasonic dissection was used for both operations (*P* = .016).^25^

Finding an association between decreased sialocele formation and use of the Sonicision would be a unique result. A sialocele is an accumulation of saliva found at a surgical site and is also commonly seen following surgeries involving the parotid gland.^26^ Previous studies (*n* = 2) that have attempted to elucidate a relationship between sialocele formation and surgical instruments have not yielded significant findings.^27,28^ Therefore, current intraoperative treatment for prevention of sialocele formation involves injecting the gland with botulinum toxin.^29-31^ This prevents glandular salivary production through inhibition of presynaptic acetylcholine release.^32^ However, the impact of surgical instrumentation in sialocele formation has been minimally explored.

The literature demonstrates an association between decreased neuropraxia and use of the Sonicision as well. When compared with electrocautery, ultrasonic dissection demonstrated a lower risk of inflicting acute and subacute neural trauma during an analysis of neural electrophysiology.^33^ In studies of the sciatic nerve in rats, statistically significant data have been found supporting the use of ultrasonic technology over electrocautery for dissection in close proximity to nerves.^34,35^ However, these studies were noted to have used monopolar cautery, which has been shown to be more risky in close proximity to nerves compared with bipolar cautery. Therefore, the demonstrated risk improvement may be partially attributable to this.

This study is predominantly limited by its sample size. At the time of our analysis, there had only been 48 partial submandibular gland removals with ultrasonic dissection completed at this center. Therefore, we were limited in the number of cases that we could use for our experimental group. This limited sample size is likely the reason for our lack of statistically significant associations between use of the Sonicision and decreased postoperative complications. Additionally, it is difficult to determine whether our results are truly caused by the switch to ultrasonic dissectors or a confounding variable such as intraoperative Botulinum toxin injection. Future expansion would likely yield more significant results, which is supported by the literature.

Future directions include a similar study that is entirely prospective with a substantially larger sample size and truly randomized groups. This would likely provide significant results, with higher power because of a greater sample size. Because there has been no previous use of ultrasonic dissection in partial submandibular gland removal, it may be useful to examine additional variables in future studies. This may include postoperative pain, edema, and operative time. Additionally, controlling for factors, such as age, gender, and BMI in statistical analysis may be useful in eliminating any additional confounding variables.^13^

The introduction of ultrasonic dissection can have a profound impact on the practice of partial submandibular gland removal in face and neck lifts. Excessive bleeding and the development of additional complications are major concerns for procedures using electrocautery devices. Descriptions of surgical techniques for this procedure frequently include warnings and instructions in case of improper hemostasis.^9,36^ Some physicians even avoid the procedure entirely, as they feel the risk of complications is too high.^37^ The authors of this manuscript demonstrate that the use of an ultrasonic dissector has the potential to alleviate these risks, with the goal of making partial submandibular gland removal a safer aesthetic procedure.

## CONCLUSIONS

This study did not demonstrate a statistically significant association between the use of ultrasonic dissection technology and a reduced incidence of hematomas, seromas, sialoceles, and neuropraxias, likely because of the lack of sufficient sample size.

## References

[1] Auersvald A, Auersvald LA. Management of the submandibular gland in neck lifts: indications, techniques, pearls, and pitfalls. Clin Plast Surg. 2018;45:507–525. doi: 10.1016/j.cps.2018.06.001 30268240

[2] Silvers AR, Som PM. Salivary glands. Radiol Clin North Am. 1998;36:941–966, vi. doi: 10.1016/s0033-8389(05)70070-1 9747195

[3] Beahm DD, Peleaz L, Nuss DW, et al Surgical approaches to the submandibular gland: a review of literature. Int J Surg. 2009;7:503–509. doi: 10.1016/j.ijsu.2009.09.006 19782158

[4] Mendelson BC, Tutino R. Submandibular gland reduction in aesthetic surgery of the neck: review of 112 consecutive cases. Plast Reconstr Surg. 2015;136:463–471. doi: 10.1097/PRS.0000000000001526 25989302 PMC4548544

[5] Iwai T, Sugiyama S, Minamiyama S, Oguri S, Mitsudo K. Clinical anatomy of feeding artery of the submandibular gland. J Craniofac Surg. 2022;33:2256–2257. doi: 10.1097/SCS.0000000000008613 35240664

[6] Garcia-Serrano G, Moñux A, Maranillo E, et al Vascular clinical anatomy of the submandibular gland. J Craniomaxillofac Surg. 2020;48:582–589. doi: 10.1016/j.jcms.2020.04.004 32389551

[7] Li L, Gao XL, Song YZ, et al Anatomy of arteries and veins of submandibular glands. Chin Med J (Engl). 2007;120:1179–1182. doi: 10.1097/00029330-200707010-00013 17637249

[8] Connell BF. Male face lift. Aesthet Surg J. 2002;22:385–396. doi: 10.1067/maj.2002.128454 19331995

[9] Bravo FG. Reduction neck lift: the importance of the deep structures of the neck to the successful neck lift. Clin Plast Surg. 2018;45:485–506. doi: 10.1016/j.cps.2018.05.002 30268239

[10] Naini FB, Cobourne MT, McDonald F, Wertheim D. Submental-cervical angle: perceived attractiveness and threshold values of desire for surgery. J Maxillofac Oral Surg. 2016;15:469–477. doi: 10.1007/s12663-015-0872-4 27833339 PMC5083688

[11] Basaran K, Comert M. Ligasure-assisted submandibular gland excision in deep plane neck lift: review of 83 patients. Plast Reconstr Surg. 2024;155:35–45. doi: 10.1097/PRS.0000000000011419 38546581

[12] Singer DP, Sullivan PK. Submandibular gland I: an anatomic evaluation and surgical approach to submandibular gland resection for facial rejuvenation. Plast Reconstr Surg. 2003;112:1150–1154; discussion 1155-1156. doi: 10.1097/01.PRS.0000076507.50053.08 12973234

[13] Hood K, Ganesh Kumar N, Kaoutzanis C, Higdon KK. Hematomas in aesthetic surgery. Aesthet Surg J. 2018;38:1013–1025. doi: 10.1093/asj/sjx236 29474524

[14] Baroudi R, Ferreira CA. Seroma: how to avoid it and how to treat it. Aesthet Surg J. 1998;18:439–441. doi: 10.1016/s1090-820x(98)70073-1 19328174

[15] Allen L, MacKay C, Rigby MH, Trites J, Taylor SM. Haemostatic devices in parotid surgery: a systematic review. J Laryngol Otol. 2021;135:848–854. doi: 10.1017/S0022215121001973 34423755

[16] Deganello A, Meccariello G, Busoni M, Parrinello G, Bertolai R, Gallo O. Dissection with harmonic scalpel versus cold instruments in parotid surgery. B-ENT. 2014;10:175–178. 25675661

[17] Ecker T, Carvalho AL, Choe JH, Walosek G, Preuss KJ. Hemostasis in thyroid surgery: harmonic scalpel versus other techniques—a meta-analysis. Otolaryngol Head Neck Surg. 2010;143:17–25. doi: 10.1016/j.otohns.2010.03.018 20620614

[18] Walker RA, Syed ZA. Harmonic scalpel tonsillectomy versus electrocautery tonsillectomy: a comparative pilot study. Otolaryngol Head Neck Surg. 2001;125:449–455. doi: 10.1067/mhn.2001.119325 11700440

[19] Leonard DS, Timon C. Prospective trial of the ultrasonic dissector in thyroid surgery. Head Neck. 2008;30:904–908. doi: 10.1002/hed.20805 18327780

[20] Kim FJ, Sehrt D, Molina WR, Pompeo A. Clinical use of a cordless laparoscopic ultrasonic device. JSLS. 2014;18:e2014.001153. doi: 10.4293/JSLS.2014.001153 PMC4216175 25392676

[21] Emam TA, Cuschieri A. How safe is high-power ultrasonic dissection? Ann Surg. 2003;237:186–191. doi: 10.1097/01.SLA.0000048454.11276.62 12560776 PMC1522135

[22] Harold KL, Pollinger H, Matthews BD, Kercher KW, Sing RF, Heniford BT. Comparison of ultrasonic energy, bipolar thermal energy, and vascular clips for the hemostasis of small-, medium-, and large-sized arteries. Surg Endosc. 2003;17:1228–1230. doi: 10.1007/s00464-002-8833-7 12799888

[23] Revelli L, Damiani G, Bianchi CB, et al Complications in thyroid surgery. Harmonic scalpel, harmonic focus versus conventional hemostasis: a meta-analysis. Int J Surg. 2016;28:S22–S32. doi: 10.1016/j.ijsu.2015.12.050 26768409

[24] Deori A, Gupta N, Gupta AK, Yelamanchi R, Agrawal H, Durga CK. A prospective randomised controlled study comparing ultrasonic dissector with electrocautery for axillary dissection in patients of carcinoma breast. Malays J Med Sci. 2021;28:97–104. doi: 10.21315/mjms2021.28.1.12 PMC7909355 33679225

[25] Lee D, Jung BK, Roh TS, Kim YS. Ultrasonic dissection versus electrocautery for immediate prosthetic breast reconstruction. Arch Plast Surg. 2020;47:20–25. doi: 10.5999/aps.2019.00759 31964119 PMC6976748

[26] Britt CJ, Stein AP, Gessert T, Pflum Z, Saha S, Hartig GK. Factors influencing sialocele or salivary fistula formation postparotidectomy. Head Neck. 2017;39:387–391. doi: 10.1002/hed.24564 27550745

[27] Chiesa-Estomba CM, Larruscain-Sarasola E, González-García JA, Sistiaga-Suarez JA, Altuna-Mariezcurrena X. Cold knife dissection and bipolar diathermy vs harmonic scalpel in parotid gland surgery for benign tumours. Acta Otorrinolaringol Esp (Engl Ed). 2020;71:93–98. doi: 10.1016/j.otorri.2019.04.00 31594557

[28] Zheng CY, Cao R, Gao MH, Huang ZQ, Sheng MC, Hu YJ. Comparison of surgical techniques for benign parotid tumours: a multicentre retrospective study. Int J Oral Maxillofac Surg. 2019;48:187–192. doi: 10.1016/j.ijom.2018.07.023 30139711

[29] Maharaj S, Mungul S, Laher A. Botulinum toxin A is an effective therapeutic tool for the management of parotid sialocele and fistula: a systematic review. Laryngoscope Investig Otolaryngol. 2020;5:37–45. doi: 10.1002/lio2.350 PMC7042652 32128429

[30] Sipkin AM, Misikov VK, Utiashvili NI. Injection of incobotulinum toxin A for the prevention of postoperative salivary fistula and sialocele. Stomatologiia (Mosk). 2021;100:55–59. doi: 10.17116/stomat202110002155 33874662

[31] Lee DJ, Lee YM, Park HJ, Lee JW, Cha W. Intraoperative botulinum toxin injection for superficial partial parotidectomy: a prospective pilot study. Clin Otolaryngol. 2021;46:998–1004. doi: 10.1111/coa.13767 33754477

[32] Lee YC, Park GC, Lee JW, Eun YG, Kim SW. Prevalence and risk factors of sialocele formation after partial superficial parotidectomy: a multi-institutional analysis of 357 consecutive patients. Head Neck. 2016;38:E941–E944. doi: 10.1002/hed.24130 25994845

[33] Chen C, Kallakuri S, Cavanaugh JM, Broughton D, Clymer JW. Acute and subacute effects of the ultrasonic blade and electrosurgery on nerve physiology. Br J Neurosurg. 2015;29:569–573. doi: 10.3109/02688697.2015.1023772 25812024 PMC4673549

[34] Tanimoto K, Khoury B, Feng K, Cavanaugh JM. Evaluation of sciatic nerve function after ultrasonic and electrocautery muscle dissection: an electromyographic study. J Neurol Surg A Cent Eur Neurosurg. 2015;76:93–98. doi: 10.1055/s-0033-1349333 23929409

[35] Carlander J, Johansson K, Lindström S, Velin AK, Jiang CH, Nordborg C. Comparison of experimental nerve injury caused by ultrasonically activated scalpel and electrosurgery. Br J Surg. 2005;92:772–777. doi: 10.1002/bjs.4948 15856482

[36] Marten T, Elyassnia D. Neck lift: defining anatomic problems and choosing appropriate treatment strategies. Clin Plast Surg. 2018;45:455–484. doi: 10.1016/j.cps.2018.06.002 30268238

[37] Rohrich RJ, Sinno S, Vaca EE. Getting better results in facelifting. Plast Reconstr Surg Glob Open. 2019;7:e2270. doi: 10.1097/GOX.0000000000002270 31624678 PMC6635200

